# The Gut Microbiota and Its Implication in the Development of Atherosclerosis and Related Cardiovascular Diseases

**DOI:** 10.3390/nu12030605

**Published:** 2020-02-26

**Authors:** Estefania Sanchez-Rodriguez, Alejandro Egea-Zorrilla, Julio Plaza-Díaz, Jerónimo Aragón-Vela, Sergio Muñoz-Quezada, Luis Tercedor-Sánchez, Francisco Abadia-Molina

**Affiliations:** 1 Department of Biochemistry and Molecular Biology II, School of Pharmacy, University of Granada, 18071 Granada, Spain; 2 Institute of Nutrition and Food Technology “José Mataix”, Center of Biomedical Research, University of Granada, Avda. del Conocimiento s/n., 18016 Armilla, Granada, Spain; alejandroegezor@gmail.com; 3 Instituto de Investigación Biosanitaria IBS.GRANADA, Complejo Hospitalario Universitario de Granada, 18014 Granada, Spain; 4 Department of Nutrition, Exercise and Sports (NEXS), Section of Integrative Physiology, University of Copenhagen, Nørre Allé 51, DK-2200 Copenhagen, Denmark; jeroav@ugr.es; 5 Departamento de Farmacia, Facultad de Química, Pontificia Universidad Católica de Chile, Santiago 6094411, Chile; chechomu@hotmail.com; 6 National Agency for Medicines (ANAMED), Public Health Institute, Santiago 7780050, Chile; 7 Cardiology Unit, Hospital Universitario Virgen de la Nieves, 18014 Granada, Spain; 8 Department of Cell Biology, School of Sciences, University of Granada, 18071 Granada, Spain

**Keywords:** cardiovascular diseases, atherosclerosis, gut microbiota, microbiome

## Abstract

The importance of gut microbiota in health and disease is being highlighted by numerous research groups worldwide. Atherosclerosis, the leading cause of heart disease and stroke, is responsible for about 50% of all cardiovascular deaths. Recently, gut dysbiosis has been identified as a remarkable factor to be considered in the pathogenesis of cardiovascular diseases (CVDs). In this review, we briefly discuss how external factors such as dietary and physical activity habits influence host-microbiota and atherogenesis, the potential mechanisms of the influence of gut microbiota in host blood pressure and the alterations in the prevalence of those bacterial genera affecting vascular tone and the development of hypertension. We will also be examining the microbiota as a therapeutic target in the prevention of CVDs and the beneficial mechanisms of probiotic administration related to cardiovascular risks. All these new insights might lead to novel analysis and CVD therapeutics based on the microbiota.

## 1. Introduction

Cardiovascular diseases (CVDs) are a group of disorders of heart and blood vessels, including hypertension (high blood pressure), coronary heart disease (disorder of the blood vessels supplying the cardiac muscle), cerebrovascular disease (disorder of the blood vessels supplying the brain), peripheral vascular disease, heart failure, rheumatic heart disease, congenital heart disease and cardiomyopathies [1]. Globally, CVD is the major cause of morbidity and mortality [2]; an estimated 17.9 million people died from CVDs in 2016, representing 31% of all global deaths. Of these deaths, 85% were due to ischemic heart disease and stroke [3]. Atherosclerosis, the precursor of myocardial infarction, or coronary artery disease, happens over periods and is related to long-term and accumulative contact to causal changeable risk factors. Different processes such as endothelial dysfunction, chronic inflammation, hyperglycemia and oxidative stress cause atherosclerosis, a complex process present in CVDs in which an inflammation response to injury is caused [4]. In atherosclerosis, the early start leading to the onset is characterized by the increase of lipids and fibrous tissue to the internal lining of arterial walls. Increased intimal thickening may eventually lead to reduced or complete occlusion of blood flow to vital organs such as the heart and brain, resulting in myocardial infarction or stroke, respectively [5].

The atherosclerosis development is defined as the formation and accumulation of foam cells within the lipid-rich subendothelial space of the affected artery. Monocytes attracted to the area will differentiate into tissue macrophages. Due to lipid metabolic pathways dysregulation, lipid-dense macrophages called foam cells are accumulated inside the arterial lining and a characteristic ‘fatty streak’ with atherogenic functions, including the release of extracellular-matrix-degrading enzymes, leading to a greater likelihood of plaque rupture and consequently blood vessel occlusion [5].

Low- and middle-income countries are affected by CVDs, out of the 17 million premature deaths (under the age of 70) due to noncommunicable diseases in 2015, 82% were in low- and middle-income countries, of which 37% were caused by CVDs [1] and happened nearly equally in men and women. Although preventive measures such as reductions in smoking, blood pressure and atherogenic lipids and advances in treatments have led to a major reduction in age-standardized death rates for CVD in high-income regions, its prevalence is rising in developing countries [1]. The factors that influence to the progress of CVD are genetic sources and epigenetic factors, environmental sources, or a combination of both [6]. On the one hand, a lesser amount of one-fifth of attributable CVD risk has been accounted for genetic determinants [7,8]. On the other hand, among environmental CVD risk factors, are contaminants (e.g., atmospheric pollution and noise), tobacco smoking, physical activity, sedentariness and what we eat, the diet. If atherosclerosis remains, it is also frequently accompanied by body weight increase, blood pressure changes, lipidemia, serum glucose, endothelial dysfunction, inflammation and thrombosis [9].

Studies in human populations and model organisms have shown that intestinal microbiota changes might be associated with CVD [10,11]. In addition, some obesity-associated comorbidities, namely type 2 diabetes (T2D) and nonalcoholic fatty liver disease (NAFLD), also exhibit perturbation of the intestinal microbiota [12,13]. Microbiota communication generates complex pathways via intestinal microbiota-generated metabolites and has been shown to disturb relevant phenotypes to CVD, covering from inflammation, insulin resistance and obesity to more direct processes similar to atherosclerosis and thrombosis susceptibility [10,14,15,16,17,18,19,20].

This review discusses the role of the human intestinal microbiota in the development of CVDs with special emphasis on atherosclerosis, some nutritional aspects, microbiota targeted therapeutics and prevention of CVD.

## 2. Relationship between Microbiota and Cardiovascular Diseases

When we talk about microbiota we refer to the ecological community of commensal, symbiotic and pathogenic microorganisms that coexist on and within an organism [21]. This comprises bacteria, archaea, fungi, protozoa and even viruses [22]; bacteria are in the spotlight due to the lack of efficient methods to study the other organisms. However, the resolution is increasing in all omics-based profiling, and the cost is decreasing as as well, which is facilitating the characterization of these other organisms [23].

The research on the human microbiota, especially gut microbiota, has come to be one of the most innovative areas when it comes to the study of different pathologies [16]. It has been demonstrated that specific microbial communities may be related with the development of several diseases like obesity [24,25], cancer [26,27,28,29], inflammatory bowel disease [30,31] and rheumatic disease [32,33]; some experiments have shown a direct connection between changes in gut microbiome and cardiovascular health and disease [15,34,35,36,37,38]. The presence of microbes in our intestine endows us with a protective milieu by inhabiting biological places that may otherwise be colonized by potentially pathogenic microorganisms [39]. Also, it is known that the microbial community exerts an effect on the host immune response and that this is an important aspect to take into consideration in the study of autoimmune diseases [40]. Besides, the microbial community has the potential for providing microbiota-derived specific molecules, such as short-chain fatty acids (SCFAs) which directly feed colonocytes and thus prevent inflammation and gut leakage [41,42,43], increase nutrient harvest [44] and alter appetite signaling [45]. The quality and quantity of each SCFA depend not only on the diet’s indigestible fraction [46] but also on a cross-feeding mechanism established in the bacterial community [47,48]. The most abundant SCFAs are acetic, propionic and butyric acids, which together represent nearly 90–95% of the SCFA present in the colon [49]. Acetate is a net product of carbohydrate fermentation of most anaerobic bacteria, while propionic and butyric acid are generated from carbohydrate or protein fermentation by a distinct subset of bacteria [50,51].

Each person can present a wide variety of microorganisms in the gut depending on several things, like their lifestyle [52,53,54,55]. It is known that the microbiota varies widely during the first year of life, then it stabilizes as a consortium that resembles that of adults [56]. The major taxa present in gut microbiota are Firmicutes and Bacteroidetes, whose magnitudes seem to remain remarkably constant over time [57,58].

In connection to the vast diversity of microbes among individuals, the nutritional status has a strong impact in gut microbiota modeling [59], to such an extent that specific diets such as those high in fats or sugars might lead to variations in the microbial population that, eventually, might facilitate the development of diseases [60]. Furthermore, exercise training is also considered a physical activity that modifies the gut microbiota composition and functional capacity [61]. Another important factor to take into account is the mental status of the individual since the presence of disorders such as anxiety [62] or depression are related with fluctuations in the gut microbiome [63]. The focus on the bidirectional association between the brain and gut microbiota, also known as gut-microbiota–brain axis, in neuropsychiatric disorders is a current field in the research of human microbiota [64,65].

### 2.1. Diet, Gut Microbiota and Cardiovascular Diseases

The gut microbiota might influence multiple metabolic and physiological processes and the modifications in these microbial structures are related with the progress of metabolic disorders such as obesity [16,66,67,68,69,70], insulin resistance [16,66,67,68,69,70] and atherosclerosis.

Foods abundant in fats (saturated, polyunsaturated and monounsaturated) are frequently copious in dietary nutrients possessing trimethylamine (TMA) moiety, such as phosphatidylcholine (PC) (lecithin), choline and L-carnitine. Mammals do not have TMA lyases, and the use of these enzymes by gut microbes, which are able to leave the C-N bond of the aforementioned nutrients, release the TMA moiety as a remaining product, so that gut microbiota are able to use these nutrients as a carbon fuel source. Portal circulation transport carries the TMA to a cluster of hepatic enzymes, the flavin-monooxygenase-3-dependent FMOs (particularly FMO3), that efficiently oxidize TMA, thus forming TMA-N-oxide (TMAO) [71,72,73,74]. 

Direct ingestion of PC, principally found in meat, poultry, fish, dairy foods, pasta, rice and egg-based dishes and the main nutritional source of choline in omnivores [75,76,77], was shown to be accompanied with increases in choline, betaine and TMAO levels [78]. Studies have shown that TMAO plasma levels are related with CVD risk [78]. Nevertheless, in other human studies, these elevated TMAO plasma levels have been independently associated with the prevalence of CVD and incident risks for myocardial infarction, stroke, death and revascularization, so more research is needed to understand the current mechanism [78,79,80,81,82,83].

Other studies have shown that L-carnitine, an another TMA-containing nutrient found almost completely in red meat, works as a nutritional precursor to gut microbial production of TMA and TMAO in mice and humans [79]. Foods abundant in cholesterol and fats, such as red meat, liver and egg yolk have the highest levels of choline and L-carnitine, and, despite the fact that many large-scale epidemiologic studies have related red meat consumption with intensified mortality and CVD risks, the association between egg ingestion and CVD risks [84,85] has shown contradictory results [84,85,86,87,88,89,90,91,92,93]. A recent study has investigated the relationship between acute consumption of egg yolk and increased plasma and urine TMAO concentrations [94]. Whereas plasma levels of choline, betaine and TMAO were related with increased CVD risk in 1876 subjects with cardiac risk evaluation [78], further analyses in cohorts exposed that the predictive significance was mostly limited to the TMAO formation, especially from choline and L-carnitine [79,95].

In a prospective clinical study employing more than 4000 subjects undertaking elective coronary angiography, high TMAO levels projected major adverse cardiac events such as death, myocardial infarction and stroke over 3 years. The major differences were observed in the patients in the upper quartile for TMAO levels with a 2.5-fold increased risk of suffering a cardiac event compared with the lowest quartile [80].

Some evidence shows a clear positive correlation of *Atopobium* to different anthropometric variables like waist circumference, weight and body mass index and also to fat and protein intake reported in a 24 h dietary recall study [96]. Additionally, metagenomic studies have demonstrated a positive correlation between *Clostridium* and TMAO formation [79] and a positive correlation between *Clostridium histolyticum/perfringens* and waist circumference, weight, body mass index and fat mass [96]; these studies suggest that the *Clostridium* species mentioned above and *Atopobium* may be considered as markers of inflammation and CVD risk.

Gut microbiota metabolites might contribute to both hypertension and inflammation [97]. Blood pressure and plasminogen activator inhibitor-1 (PAI-1) levels have been associated to the gut microbiota composition in overweight and obese pregnant women. The butyrate-producing genus *Odoribacter* abundance has been oppositely correlated with systolic blood pressure. Butyrate production capacity is lower and PAI-1 concentrations higher in obese pregnant women. In addition, PAI-1 levels have been conversely correlated with the butyrate kinase expression and *Odoribacter* abundance [97]. A recent meta-analysis from prospective studies has concluded that elevated TMAO concentrations and its precursors were related with increased risks of major adverse cardiovascular events and all-cause mortality independently of traditional risk causes [98]. After the administration of wine with polyphenols, the authors reported a significant increase of 4-hydroxyphenylacetate in a healthy cluster. Other results in humans have shown gut microbiota responsive phenotypes to wine polyphenols intervention [99].

Preliminary results of the Prevention with Mediterranean Diet (PREDIMED) study have shown that baseline plasma concentrations of choline and hydroxyproline were associated with higher CVD risk independent of traditional risk factors, while no significant association between plasma concentrations of TMAO and CVD was found. The plasma concentrations of choline and hydroxyproline were associated with a 2.13-fold higher risk of CVD across extreme quartiles and a 1.99-fold higher risk of stroke, and baseline betaine/choline ratio was inversely associated with CVD. Compared to participants with a score below the median and randomized to the Mediterranean diet, the hazard ratio of developing CVD was 2.56 for participants with a gut microbiota score above the median and randomized to the control group [100]. TMAO levels have also been correlated with brain-type natriuretic peptide and associated with both heart failure severity and heart failure mortality [101].

Three recent meta-analyses have established that elevated TMAO blood levels are related with increased CVD risks and all-cause mortality [98,102,103], nevertheless, some criticism exists about the TMAO and CVD relationship because fish could contain high concentrations of TMAO and TMA [104]. However, fish consumption is related with heart health [105,106,107]. Also, there is a study without association with measures of atherosclerosis and TMAO [108]. More randomized clinical trials and larger studies are needed to clarify if TMAO is a marker or mediator in CVD. The contribution of gut microbiota in our health, immune function and disease development, continue to be generally unknown areas. Other compounds such as intestinal-derived endogenous endotoxins, e.g., lipopolysaccharides [109], indoxyl sulfate [110] and para-cresyl sulfate [111], have been suggested to play important metabolic roles in conditions ranging from atherosclerosis to cardio-renal dysfunction [112,113,114].

A recent study in mice showed that the products generated by the intestinal microbiota such as SCFAs, secondary bile acids, endotoxins and tryptophan metabolites, are often altered in diets rich in fat (coconut oil and soybean oil) and low in fiber and would then impact L-cell glucagon-like peptide (GLP)-1 secretion [115].

### 2.2. Microbiota and Cardiovascular Diseases

It has been investigated whether metabolites derived from microbiota could influence the composition of fluids within the human body. such as blood and urine, and whether they may regulate fat absorption and bile acid/cholesterol metabolism among other physiological functions [116]. The gut microbiota can possibly affect host blood pressure through multiple mechanisms. Bacteria belonging to *Bifidobacterium*, *Lactobacillus*, *Streptococcus* and *Escherichia* genera can produce neurotransmitters within the autonomic nervous system [117]. Modifications in the prevalence of these bacteria might change the vascular tone and contribute to the hypertension development or other CVD [97,118,119,120]. Metabolomics approach have shown that dietary lipid phosphatidylcholine and its metabolites betaine, TMAO and choline are risk factors for CVD [78]. One study that comprised three different groups of men distributed according to the European Society of Hypertension criteria based on 24-h ambulatory blood pressure measurements supports this since the results obtained indicated a positive correlation between blood pressure and SCFA levels [121]. Another study on patients with prehypertension and stage 1 hypertension found that hippurate, phenylacetylglutamine and 4-cresyl sulfate found in urine were related with blood pressure [122].

A systematic review of human studies has reported that the *Faecalibacterium*, *Bifidobacterium*, *Ruminococcus* and *Prevotella* abundances are conversely linked to different low-grade inflammation markers such as high sensitivity C-reactive protein and interleukin (IL)-6. The existing relationships between the gut microbiota and low-grade inflammation markers in humans and the benefit of a therapeutic strategy to prevent and treat atherosclerotic CVD considering the gut microbiota and its relation with the innate and adaptive immune system [123] underline the importance of the investigation into the human gut microbiota as a potential diagnostic tool.

It has been observed how the bacteria located in the oral cavity might be related to CVD [124]. A study in patients admitted for acute coronary syndrome showed a higher subgingival bacterial load when compared to controls; the species that were mostly increased in this study were *Streptococcus intermedius*, *S. sanguis*, *S. anginosus*, *Tannerella forsythensis*, *T. denticola* and *Porphyromonas gingivalis*. Hence, these species could be risk issues for the development of acute coronary syndrome [125]. Furthermore, a possible association between *Actinobacillus actinomycetemcomitans* present in the oral cavity and both coronary heart disease and stroke has been described after several sero-epidemiologic studies [126,127].

### 2.3. Microbiota and Atherosclerosis

In mice, a choline-rich diet increased TMAO levels and atherosclerosis, depending on gut microbiota activity, as shown by broad-spectrum antibiotics treatment [128]. On the other hand, gut microbiota influences the host inflammatory response, altering endothelial function, which can influence host blood pressure. SCFAs production by the gut microbiota is associated with hypertension, as a result of the influence of SCFA on vascular tone [43,109,129].

Another study on men with atherosclerotic plaque on the carotid wall who consumed a drink with high numbers of *Lactobacillus plantarum* (DSM9843), showed an increased bacterial diversity compared to the placebo group as well as a decrease in the concentration of some SCFA [130], suggesting that the consumption of this strain might be a strategy to favor the intestinal diversity in patients with atherosclerotic plaque on the carotid wall.

Recent studies have directly related high levels of TMAO with an increase in cardiovascular risk and its severity [131,132]. Accordingly, TMAO levels have been correlated both with atherosclerotic plaque size and cardiovascular events [78]. Other research studies have observed that atherosclerotic plaques contain bacterial DNA, and the bacterial taxa observed were also present in the gut and oral microbiota of the same individuals [133,134]. Several epidemiological studies have associated periodontal disease and CVD [135,136,137]; an oral microbiota role in the CVD pathophysiology has also been studied [133,137,138,139].

In addition, metagenomic analyses have shown that microbial composition is altered in patients with unstable compared with stable plaques; unstable plaques are related with reduced *Roseburia* fecal levels and both increased theoretical capacity of the microbiome to produce proinflammatory peptidoglycans and reduced production of anti-inflammatory carotenes [11].

Other examples of the relationships between microbiota and atherosclerosis are the administration of metformin and whole grains; metformin is a biguanide antidiabetic drug widely used in adults that have shown to exert positive effects to fight against CVD risk and that might be used safely in patients with heart failure and even reduce its occurrence or mortality, not only by direct effects [140], but also because of the possible effects produced through gut microbiota remodeling [141]. Also, the diet seems to be a potential therapy to diminish the risk of CVD since a study on a specific population of Danish adults showed that a diet abundant in whole grain compared to refined grain reduces body mass and systemic inflammation [142], which are risk factors to a bad prognosis of CVD.

### 2.4. Other Microbiota Aspects Related to Cardiovascular Diseases

One of the most studied pathogen-associated molecular patterns (PAMPs) concerning cardiovascular function and the increase in CVD risk is lipopolysaccharide (LPS), a Gram-negative bacterial cell wall component [143,144]. Circulating LPS is raised in at-risk individuals and predicts future CVD [145,146,147]; accordingly, administration of low-doses of LPS induces vascular inflammation and atherosclerosis in experimental animals [148,149]. Another significant PAMP is the peptidoglycan that can trigger the nucleotide-binding oligomerization domain (NOD) receptors. NOD receptors can identify bacterial determinants once they are phagocytosed by macrophages and dendritic cells. NOD2-deficient mice fed with a high-fat diet have shown increased bacterial translocation and insulin resistance [150]. Additionally, human genetic and mouse knockout studies have investigated the role of NOD2 in atherosclerosis [151,152].

#### 2.4.1. Microbiota, Choline and Homocysteine Cycle

Recently, a study reported that microorganisms in the gut microbiota hydrolyze PC to obtain choline for downstream metabolism [153,154]. A previous study reported that gut microorganisms can anaerobically convert choline to TMA, which is further metabolized by the host to TMAO [78]. Analysis in gnotobiotic mice has revealed that specific bacteria might increase the TMAO formation [155].

Choline is an essential nutrient that is usually grouped within the vitamin B complex. Choline and its metabolite betaine are methyl donors along with folate, and are metabolically linked to transmethylation pathways including synthesis of the CVD risk factor homocysteine [78]. Deficiency in both choline and betaine has been suggested to produce epigenetic changes in genes linked to atherosclerosis [156,157], and acute choline and methionine deficiency in rodent models causes lipid accumulation in liver (steatohepatitis), heart and arterial tissues [158]. Homocysteine, a sulfhydryl-containing amino acid produced via demethylation of methionine and essential for intravascular metabolism [159], has been supposed as a reasonable risk issue for the atherosclerotic vascular disease leading to CVD and stroke [160]. Highly elevated homocysteine levels in genetic hyperhomocysteinemia are pathogenic to the vascular system, and homocysteine, at comparably high levels, also exerts proinflammatory effects on vascular cells in vitro [161].

Higher dietary choline intake was associated with a lower risk of incident ischemic stroke in African-American participants; also, higher dietary betaine intake was associated with a nonlinear higher risk of incident coronary heart disease [162].

#### 2.4.2. Vitamin B-Complex and Microbiota

Commensal bacteria are suppliers and consumers of B vitamins and vitamin K. While dietary B vitamins are generally absorbed through the small intestine, bacterial B vitamins are produced and absorbed mainly through the colon [163,164], showing that dietary and gut microbiota-derived B vitamins are probably controlled differently by the human body.

In a prospective study with Korean men, the authors found that higher dietary intake levels of vitamin B6 were associated with a reduced CVD risk [165]. Vitamin B3 might increase all-cause mortality, which was probably associated with its adverse effects on glycemic response [166,167].

Vitamin B9 and B-vitamin complex reduced risk for stroke, and vitamin B9 reduced risk for total CVD events. There was no evidence of a reduction of CVD risk with any other vitamins or supplements, and no supplements reduced mortality [168]. A recent meta-analysis found that vitamin B9 supplementation significantly reduced the risk of stroke in patients with CVD [169].

#### 2.4.3. Low-Grade Inflammation

Evidence exists that inflammation and oxidative stress are influenced by the diet, and it may, therefore, be possible to reduce or delay the effects of age-related changes in these parameters through appropriate dietary intervention and/or use of nutraceutical dietary supplements [170].

Some studies have investigated the relationship between gut microbiota and markers of chronic low-grade inflammation in humans. An opposite association among *Prevotella* and inflammatory markers and an increased abundance of certain *Prevotella* species were associated with low-grade inflammation in systemic diseases, such as rheumatoid arthritis [171]. In addition, *Prevotella* abundance was inversely associated with LPS and high sensitivity C-reactive protein. Furthermore, individuals with obesity have a lower abundance of *Prevotella* species in their gut [171].

The RISTOMED project is an open-label study that investigated the diet as a means to improve health-related quality of life for older people and to prevent aging-related diseases and also the concomitant administration of VSL#3, a mixture of probiotic strains, in the possible reduction of high-sensitivity C-reactive protein plasma concentration and microbiota changes due to the fact that this protein is defined as a cardiovascular risk in this population by the American Heart Association [170]. Changes in the aforementioned outcomes were observed in a subgroup analysis in participants with low-grade inflammation. The RISTOMED diet plus VSL#3 administration has shown a reduction in high-sensitivity C-reactive protein and also an increase in *Bifidobacterium* species [170,172]. Further analyses with more participants in the study have shown similar results in high-sensitivity C-reactive protein and microbiota [173]. Similar studies, involving the administration of probiotics in elderly human trials have shown no effects on inflammatory outcomes [174,175,176,177,178], augmented levels of fecal prostaglandin E_2_ [179] and diminished plasma endotoxin, the soluble cluster of differentiation 14 and LPS binding protein levels [180].

Recently, Gil-Cruz et al. reported that mimic peptides from commensal bacteria can promote inflammatory cardiomyopathy in genetically susceptible individuals [181].

In brief, several studies have established the relationship between homocysteine, PAMP, low-grade inflammation, microbiota and CVD. In this regard, further studies are needed to determine the specific factors and the underlying mechanism in the progression and prevention of CVD.

## 3. Microbiota-Targeted Therapeutics

### 3.1. Physical Activity, Microbiota and Cardiovascular Diseases

In the last 10 years, it has been observed that there is a possible relationship between the intestinal microbiota and the cardiovascular system [11,144,182,183]. Human cardiometabolic health has been related with variations in the gut microbiota composition (dysbiosis) [184]. Kelly et al. reported that subjects with a high lifetime burden of CVD risk factors had less microbial wealth compared to those with a low lifetime burden, identifying a high number of Bacteroidetes and Firmicutes [185].

The benefits of regular physical activity against cardiovascular problems are widely known. Recent studies have revealed how physical exercise affects gut microbiota [186,187,188,189,190]. Increased levels of Bacteroidetes and decreased of Firmicutes were observed in obese adults who had moderate to severe aerobic exercise for 10 weeks [191]. Although no direct evidence supports the idea that physical exercise prevents atherosclerotic CVD through changing the gut microbiota and by improving systematic inflammation, many studies have supported this hypothesis [186]. Zuheng Liu et al. reported that the changes in the gut microbial organization that are produced by physical exercise are associated with cardiac function in myocardial infarction mice [192].

It is known that voluntary running exercise modifies the microbiota composition of the cecum and increases the n-butyrate concentration in the cecal content [189]. Butyrate is one of the three most important SCFAs, and several studies have shown that it may have effects on cardiovascular function [97,144,183]. Nevertheless, more studies are needed to explore the principal physiological mechanisms that relate regular exercise to SCFA levels and its effect on blood pressure and inflammation.

### 3.2. Probiotic Administration, Microbiota, Bile Acids and Cardiovascular Diseases

It has been demonstrated that probiotics can affect the structure of gut microbiota and the interaction with the microbial community and the host health through different mechanisms [12,193,194,195]. These effects are mediated by the direct or indirect action of probiotics and can involve the modulation of the immune system or that of remote organs like the brain and liver due to the production of metabolites finally localized in these organs [193,196,197,198,199].

Obesity is one of the primary risk factors for the development of CVD and presents a major risk for T2D, hypertension and hyperlipidemia and predisposes to coronary heart disease [200,201]. Hypercholesterolemia is directly associated with the prevalence of ischemic heart disease in both men and women [202]. Dietary modifications are the first line of treatment and offer an effective means of reducing blood cholesterol levels. However, the low rate of patient dietary compliance means that drug administration is one of the most effective treatments to control plasma cholesterol, triacylglycerols and blood sugar levels.

There is evidence supporting that probiotics can improve some parameters of the risk factors of CVD, like obesity. A recent systematic review reported that specific strains from *Lactobacillus* and *Bifidobacterium* have been generally used as probiotic treatment in well-established animal models of obesity [203] and in blood lipid index, T2D and hypertension [200,201,204,205,206].

The potential probiotic mechanisms related to the hypocholesterolemic effect could involve active bile salt hydrolase (BSH), cholesterol co-precipitation with deconjugated bile salts, bacterial cell membrane assimilation and incorporation of cholesterol, conversion of cholesterol to coprostanol through the cholesterol reductase enzyme and SCFA production [207]. The BSH increased fecal excretion of free bile acids, preventing their reabsorption and compensatory increased use of cholesterol to produce bile acids, which could lead to a reduction in the cholesterol present in serum. SCFAs can inhibit the hepatic activity of the 3-hydroxymethyl-3-glutaryl-CoA reductase, the hepatic enzyme in the process of hepatic cholesterol synthesis, while the propionate can stimulate the bile salts hepatic synthesis through increasing the activity of 7α-hydroxylase [208].

The antihypertensive effects of probiotics have been related to their metabolites; some studies have reported specific bioactive tripeptides. These compounds have an angiotensin-converting enzyme (ACE)-inhibitory properties [209]. However, other studies have related these bioactive peptides with up to 12 peptides in length of fermented milk containing probiotics with similar antihypertensive effects [210].

Probiotics improve T2D symptoms, glucose biomarkers and insulin resistance by restoring homeostasis of gut microbiota. Furthermore, a meta-analysis suggests that the supplementation of probiotics has a modest effect on the serum level of fasting blood sugar as well as oxidative stress biomarkers [211]. Mechanisms that have been proposed are as follows: improved intestinal integrity, decreased systemic lipopolysaccharide levels, decreased endoplasmic reticulum stress and improved peripheral insulin sensitivity [204,212]. Data from clinical studies and animal models have shown a reduction in lipopolysaccharide translocation, endotoxemia and inflammation, reducing stimulation of the proinflammatory genes like tumor necrosis factor alpha (TNF-α), IL-6 and IL-1β [204,213].

In contrast, negative results have been found in other studies and meta-analyses regarding the effectiveness of probiotics in diarrhea prevention in children [214], adults [215] and the elderly [216,217]. A consistent and sufficient probiotic consumption might produce a number of health benefits including reducing CVD risk factors [201], nevertheless, further studies in different models are necessary for a better understanding of the beneficial mechanisms of probiotic administration in CVD risk alone or accompanied by foods; certainly, probiotics act in a strain-specific manner and are often used as coadjuvant therapy. Likewise, recent individual studies and meta-analyses should be, mainly due to the different probiotic strains used, carefully interpreted.

### 3.3. Fecal Microbiota Transplantation

Fecal microbiota transplantation (FMT) has become popular in recent years. FMT is the transplantation of functional bacteria from feces of healthy donors into the gastrointestinal tract of patients to repair the balance of the intestinal microbiota [218]. The process involves the collection of filtered stools collected from either a healthy donor or from the recipient himself (autologous FMT) at a time point before initiation of disease and associated dysbiosis and its installation into the intestinal tract of a patient suffering from a certain medical condition [219]. FMT is effective in the treatment *Clostridium difficile* infection (CDI) in humans [220,221]. The first report of FMT application in the treatment of CDI dated from 1983 [222]; in 2010, the United States Infectious Diseases Society of America and Society for Healthcare Epidemiology of America recommended FMT as a treatment plan for CDI in their clinical guidelines [223]. Recently, some studies have shown that there is a very strong potential application for FMT in the field of cardiometabolic disorders [224,225], such as atherosclerosis, metabolic syndrome and T2D [226]. However, FMT is currently restricted due to its associated risks, including the possible transfer of endotoxins or infectious agents that could cause new gastrointestinal complications [227,228].

Further studies are needed to examine whether FMT might be extended to other facets of cardiometabolic disorders. Instead of fecal contents, the transplantation of only a defined group of bacteria may be a rational alternative to FMT. Also, further research is needed to better define the optimal fecal microbial preparation, dosing and method of delivery.

### 3.4. Personalized Nutrition

Evidence shows that variations occasioned by dietary interventions in host metabolism are person-specific [229], and, because not all individuals respond to diet in the same way (e.g., weight gain, postprandial glucose, etc.), personalized nutrition is a new therapeutic possibility for prevention and control of disease [230]. Recent studies of cohorts have revealed great differences in post-meal glucose levels between individuals eating the same mealtimes [229,231].

Healthy participants who exhibited enhanced glucose metabolism following barley kernel-based bread (BKB) consumption were related with a greater *Prevotella* abundance [232]. Another study in humans has reported that the whole grains ingestion induced anti-inflammatory responses and blood glucose level changes of different magnitudes; participants with greater blood IL-6 improvements had higher *Dialister* levels and lower Coriobacteriaceae species in their stools [233]. Furthermore, on a calorie-restricted diet, overweight and obese adults with higher levels of baseline *Akkermansia muciniphila* presented a greater improvement in insulin sensitivity and lipid metabolism, as well as a greater reduction in body fat [234]. Another cohort including 800 overweight or obese nondiabetic individuals showed high interpersonal variability in the postprandial glycemic response to identical foods, which was predicted accurately by different factors, i.e., the gut microbiome, dietary habits, blood parameters and anthropometrics, using a machine learning approach [229]. These results concluded that microbiota-based nutrition can be used to expect variable clinical phenotypes in metabolic syndrome as well as gastrointestinal disorders; individuals can then be classified into responders and nonresponders based on different outcomes such as dietary components, age, serum parameters and the microbiome, all contributing to personalized predictions [230].

**Figure 1:**
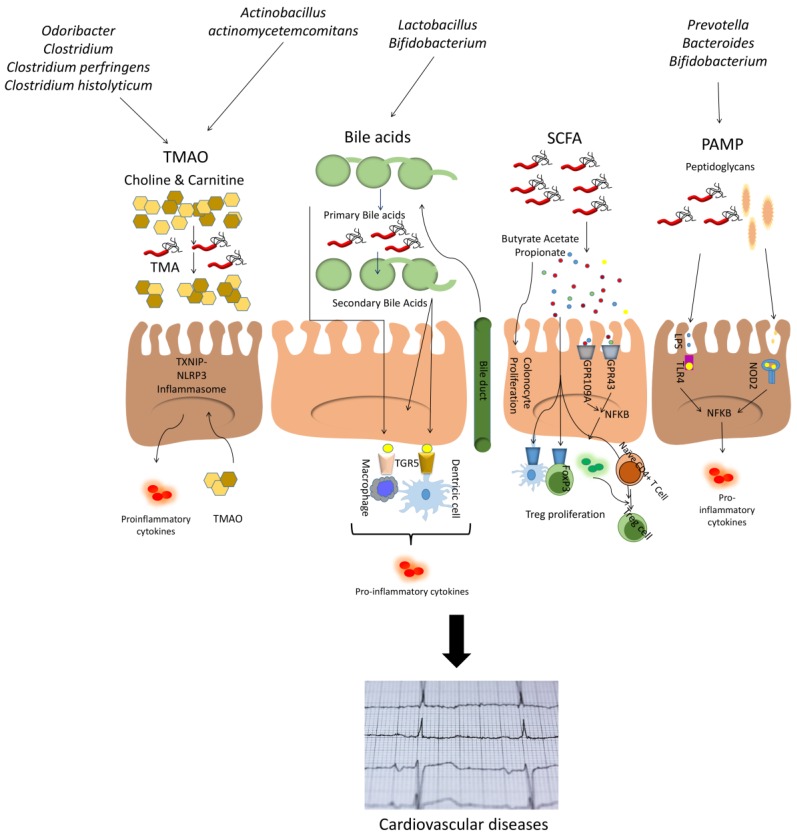
Schematic representation of the relationships between microbiota and cardiovascular disease (CVD). Abbreviations. LPS, lipopolysaccharide; PAMP, pathogen-associated molecular patterns; NFκB, nuclear factor kappa-B; SCFA, short-chain fatty acids; TLR, toll-like receptor; TMAO, trimethylamine N-oxide.

Interindividual variability regarding the efficacy of certain nutrients in optimizing an individual’s health and the identification of factors that give to an individual’s response to diet, as well as developing methods of personalizing dietary references, are shown to be critical [235]. Figure 1 summarizes the relationship between microbiota and related metabolites and CVD.

## 4. Prevention of Cardiovascular Diseases

The balance between pathogenic and nonpathogenic microorganisms in the gut is critical to maintaining the lifelong health of humans. As previously mentioned, the diet is an external factor that influences the gut microbiota composition. Different studies have analyzed diets and their implications with the gut microbiota and the prevention of CVD. The Mediterranean diet, based on the regular ingestion of plant foods, the moderate consumption of fish, seafood and dairy, a low-to-moderate alcohol (mostly red wine) intake, balanced by a comparatively limited use of red meat and other meat products, with olive oil being the main source of fat consumed in this diet, is nowadays universally recognized as beneficial to health by medical professionals and could be an emerging medical prescription [236] based on the reduction incidences of insulin resistance, hypertension, CVD, T2D and metabolic syndrome [236]. Other important diets for the prevention of CVD are plant-based diets, which are characterized by high consumption of seeds, cereals, fruit, berries, nuts and vegetables. Both diets are important sources of fibers and bioactive compounds, which are metabolized by microbes to produce different metabolites [237] such as acetate, propionate and butyrate, which are involved in suppressing inflammatory responses. The mechanisms by which these diets exert their beneficial effects remain to be elucidated, but their bioactive food components such as unsaturated fatty acids [238], complex carbohydrates and fibers [237] and polyphenols [239] are very implicated.

Unsaturated fatty acids, in particular n-3 polyunsaturated fatty acids, are generally considered cardiovascular-protective. Fish oil is the main source of animal oil whereas flaxseed oil is obtained from plants [238]. Both fish oil and flaxseed oil could modulate gut microbiota and enhance the microbial production of SCFAs, with fish oil being more effective than flaxseed oil in promoting the growth of SCFA-producing bacteria and lowering microbial generation of LPS; both oils are implicated in the reduction of TMAO, with fish oil being the most effective in exacerbating atherogenesis [238]. Beta-glucan, a natural polysaccharide from the plant cell walls, belongs to one of the dietary fiber fractions considered to be a prebiotic which stimulates the growth of beneficial intestinal bacteria [240], produces SCFA [237] and reduces cholesterol and glucose concentrations in the blood, all of which reduces the risk of CVD and diabetes.

Polyphenols, mainly founded in the Mediterranean and plant-based diets, are a group of phytochemicals abundant in the human diet and considered to be very important in the prevention of diseases by their ability to modulate the microbiota. In animal models, polyphenols increase bacteria that cause SCFA production and decrease bacteria that produce LPS. The most important polyphenols groups, mainly founded in fruits, are flavonoids, flavones and flavonols [239]. Accordingly, it has been shown that the intake of whole fruits is a good strategy for the prevention of diseases by increasing the growth of beneficial bacteria (i.e., *Bifidobacterium* and *Lactobacillus*) [241], which is in agreement with previous research with pomegranate polyphenol extracts [242] and in animal studies [239].

In conclusion, the consumption of a healthy diet based on unsaturated fatty acids, fruits and vegetables is the best strategy in the prevention and treatment of diseases that are modulated by gut microbiota.

## 5. Further Directions and Perspectives

The gut microbiota influences drug responses altering both pharmacodynamics and pharmacokinetics. Activity from the gut microbiota can thus result in altered drug pharmacokinetics, activation of prodrugs and the unwanted formation of toxic metabolites or inactivation of drugs [243]. Each patient displays significant variations in response to treatment and drug-associated injurious effects, which results in considerable variations in morbidity and mortality [244,245,246].

Personalized nutritional approaches can be established to change an individual’s microbiome and further develop the response to a specific diet. The future of personalized nutrition will allow for the rational design of diets. A prior step would include the individual analysis of the microbiome, the prediction of particular responders and nonresponders and the identification of beneficial foods for the different microbiome types and desired outcomes. In relation to CVD and atherosclerosis, the personalized diet recommendation would depend on the patient microbiota, the TMAO blood levels and the family history [230].

A greater understanding of the interactions between the patient microbiome and the response to treatments will be fundamental for the improvement of CVD therapies and the development of novel approaches targeting the microbiota in CVDs.
